# The Impact of Left Ventricular Hypertrophy and Diastolic Dysfunction on Outcome in Intracerebral Hemorrhage Patients

**DOI:** 10.1155/2013/898163

**Published:** 2013

**Authors:** Karen C. Albright, Joshua M. Burak, Tiffany R. Chang, Aimee Aysenne, James E. Siegler, Laurie Schluter, Sharyl R. Martini, Amelia K. Boehme, Sheryl Martin-Schild

**Affiliations:** 1 Health Services and Outcomes Research Center for Outcome and Effectiveness Research and Education (COERE), University of Alabama at Birmingham, Birmingham, AL 35294-4410, USA; 2 Center of Excellence in Comparative Effectiveness Research for Eliminating Disparities (CERED) Minority Health & Health Disparities Research Center (MHRC), University of Alabama at Birmingham, Birmingham, AL 35294-4410, USA; 3 School of Public Health, Department of Epidemiology, University of Alabama at Birmingham, Birmingham, AL 35294-0022, USA; 4 Department of Medicine, Cardiology Section, Tulane University School of Medicine, New Orleans, LA 70112, USA; 5 Division of Neurosciences Critical Care Medicine, Johns Hopkins University, Baltimore, MD 21287, USA; 6 Stroke Program, Department of Neurology, Tulane University School of Medicine, New Orleans, LA 70112, USA; 7 Department of Neurology, University of Cincinnati College of Medicine, Cincinnati, OH 45219, USA; 8 Stroke Program at Tulane University Hospital, Department of Neurology, Suite 1000, 1440 Canal Street, TB-52, New Orleans, LA 70112-2715, USA

## Abstract

**Background:**

The objective of this study was to determine the prevalence of LVH and DD in patients presenting with supratentorial deep ICH and to determine if the presence of LVH or DD was an independent predictor of initial ICH volume, hematoma expansion, or poor outcome.

**Methods:**

A cross-sectional study was performed on ICH patients who presented from 7/2008 to 12/2010. Cases were excluded if ICH was traumatic, lobar, infratentorial, secondary to elevated international normalized ratio, suspicious for underlying structural malformation, or where surgical evacuation was performed. Logistic and linear regressions were used to assess the ability of LVH to predict ICH imaging characteristics and patient outcomes.

**Results:**

After adjusting for use of hemostatic agents, LVH was not a significant independent predictor of initial ICH volume (*P* = 0.344) or 33% volume expansion (*P* = 0.378). After adjusting for age, infectious complications, and use of hemostatic agents, LVH was not a significant independent predictor of poor functional outcome (*P* = 0.778). Similar results were seen for DD.

**Conclusion:**

In our sample, patients with deep ICH and LVH were more likely to develop IVH, but LVH was not a significant independent predictor of initial ICH volume, hematoma expansion, or poor short-term outcome.

## 1. Introduction

Intracerebral hemorrhage (ICH) accounts for 10% to 15% of strokes [1, 2]. With an estimated 30-day mortality rate greater than 40% and fewer than 1 in 5 survivors functionally independent at 6 months, ICH is more likely to result in death and disability than ischemic stroke [3–6].

Epidemiologic evidence suggests that the pathophysiology of spontaneous ICH differs for lobar and deep ICH [7]. Lobar ICH, frequently seen in the elderly, is often presumed to be the result of amyloid angiopathy, whereas deep ICH, such as this seen in the basal ganglia, is attributed to a modifiable risk factor—hypertension (HTN). The majority of hypertensive intracerebral hemorrhages are located in deep supratentorial regions [8–13].

In addition to being the single largest risk factor for ICH, hypertension is the primary risk factor for cardiac disease [14]. Hypertensive end organ damage in the heart (i.e., hypertensive heart disease) is prevalent with reported rates of left ventricular hypertrophy (LVH) in hypertensive patients of 36%–41% [15]. In patients with resistant hypertension, these rates range from 55% to 75% [16]. While LVH has been shown to be a significant independent predictor of myocardial infarction, stroke, and cardiovascular death in the general population [17,18], patients with coronary disease [19] and hypertensive patients [17,18], it has not been clearly established as a predictor of poor outcome in ICH patients.

HTN is common in the setting of ICH, but little is known about the prevalence and impact of the severity of chronic HTN, as evidenced by LVH and diastolic dysfunction (DD), in patients with hypertensive ICH [20–22]. The primary objective of this study was to determine the prevalence of LVH and DD in patients presenting with ICH presumed to be secondary to HTN (deep ICH). The secondary objectives were to determine if the presence of LVH or DD is an independent predictor of initial ICH volume, hematoma expansion, or poor outcome in patients with deep ICH.

## 2. Methods

The greater New Orleans area has a population of 1.2 million people, with 34% African American. Tulane Medical Center is a for-profit hospital with an 8-bed stroke unit, staffed by Tulane University College of Medicine physicians. A cross-sectional study was performed on patients who presented to the Tulane Emergency Department (ED) between July 2008 and December 2010 with imaging that confirmed intracerebral hemorrhage (ICH, ICD-9 code 431). HTN was treated per the AHA/ASA guidelines for the early management of patients with ICH [23]. Patients were included if greater than 18 years of age. Participants in whom ICH was traumatic, lobar, infratentorial, and suspicious for underlying structural malformation as the cause of ICH were excluded as they did not represent the sample of interest. In addition, ICH cases secondary to elevated international normalized ratio (INR) or where surgical clot evacuation was performed were excluded, as medical and surgical intervention has been shown to affect ICH volume.

Baseline demographics, stroke severity on admission as measured by the National Institutes of Health Stroke Scale (NIHSS) and ICH scores, past medical history, home medication use, imaging, and laboratory findings were collected. Time from last seen normal (LSN) to initial head CT was collected as a categorical variable (<3 hours, 3–6 hours, >6 hours). Repeat head CT was performed within 24 hours of admission. Patient outcomes were assessed using discharge disposition and the modified Rankin Scale (mRS) score at discharge. Poor discharge disposition was defined as not being discharged to home or inpatient rehabilitation. Discharge mRS scores of 4–6 were used to define poor discharge mRS. This study was approved by the Tulane University Institutional Review Board.

Categorical variables were compared using Chi-square or Fisher's exact, where appropriate. Continuous variables were compared using independent samples *t*-test or Mann-Whitney *U* test, where appropriate. Logistic and linear regressions were used to assess the ability of left ventricular hypertrophy and diastolic dysfunction to predict ICH characteristics on imaging and patient outcomes. Given their effect on ICH characteristics, it was determined a priori to adjust for use of hemostatic agent on regressions involving ICH characteristics on imaging. In a similar manner, the decision was made to adjust for age, presence of infectious complication, and use of hemostatic agent on regressions involving patient outcomes.

## 3. Results

During the 30-month period, 121 ICH cases were screened. Cases were excluded due to infratentorial location (*n* = 20), lobar location (*n* = 28), elevated INR (*n* = 3), surgical clot evacuation (*n* = 2), and lack of available transthoracic echocardiogram (TTE) data (*n* = 19). Participants were young, with an average age of 58. More than 60% of cases were males; 77% were black. While the median stroke severity was moderate (NIHSS 15), in general, patients presented awake and alert (median GCS 14). The majority of patients (85%) reported a history of hypertension; less than half (47%) reported taking medication for their HTN at home. Mean systolic blood pressure (SBP) on presentation was 194 with a mean diastolic blood pressure (DBP) of 110. The prevalence of left ventricular hypertrophy (LVH) was 38%, whereas the prevalence of diastolic dysfunction (DD) was 68% in this population. The median ICH score for deep supratentorial ICH patients was 1, with median initial ICH volume of 12 milliliters (mL). Nearly half of the patients (43%) presented with intraventricular extension (intraventricular hemorrhage, IVH). More than 1/3 of patients (36%) presented with hydrocephalus.

In an effort to address a potential source of bias, patients meeting inclusion criteria that did not have a TTE performed were compared to those with a TTE. As shown in Table 1, there were no statistically significant differences in baseline characteristics in these two groups. Of the 47 patients with TTE, time from LSN to initial CT was available on 45. More than half (57.8%) of initial CTs were performed within 3 hours, with 11.1% in 3–6 hours, and 31.1% more than 6 hours from symptom onset. All patients had repeated head CT within 24 hours of admission.

Table 2 compares the demographics, baseline stroke severity and blood pressure on presentation, ICH characteristics, and TTE findings in patients with LVH to patients without LVH. In our sample, we found only two significant differences: (1) mean SBP was higher in patients with LVH (213 versus 183 mm Hg, *P* = 0.005) and (2) the size of the left ventricle was significantly larger in patients with LVH (148 versus 90 g/m^2^, *P* = 0.003) compared to patients without LVH. No significant differences were found in the proportion of patients receiving initial head CT <3 hours, 3–6 hours, and >6 hours when patients with LVH were compared to patients without LVH. These variables were also compared in patients with DD to patients without DD. No significant differences were detected between groups.

Medical interventions, in-hospital complications, changes in ICH characteristics, and short-term outcomes are shown in Table 3. When compared to patients without LVH, patients with LVH were almost three times more likely to develop new IVH on follow-up neuroimaging when compared to patients without LVH (OR 2.9, 95% CI 2.0–4.4, *P* = 0.050). There were no significant differences found with regards to interventions, complications, or outcomes. In a similar manner, no significant differences were detected in patients with DD when compared to patients without DD.

After adjusting for use of hemostatic agents, LVH was not a significant independent predictor of initial ICH volume (*P* = 0.344), IVH on presentation (*P* = 0.729), 10 cc ICH expansion (*P* = 0.534), 33% volume expansion (*P* = 0.378), or new IVH on follow-up neuroimaging (*P* = 0.997). Similarly, the presence of DD was not a significant independent predictor of initial ICH volume (*P* = 0.747, IVH on presentation (*P* = 0.788), 10 cc ICH expansion (*P* = 0.778), 33% volume expansion (*P* = 0.974), or new IVH on follow-up neuroimaging (*P* = 0.237).

After adjusting for age, presence of infectious complication, and use of hemostatic agent, neither the presence of LVH nor the presence of DD was a significant independent predictor for length of stay (*P* = 0.351, *P* = 0.343), poor disposition at the time of discharge (*P* = 0.590, *P* = 0.822), and poor functional outcome (*P* = 0.778, *P* = 0.836). The presence of LVH was associated with a nonsignificant higher likelihood that patients would require a minimum of three blood pressure medications at the time of discharge (OR 7.9, 95% CI .90–71.4, *P* = 0.062). This association was not observed for DD (OR 2.0, 95% CI .45–8.9, *P* = 0.364). Despite this, only 13.3% of patients with LVH achieved a goal BP of 140/90 at the time of discharge, as compared to 61.5% of patients without LVH (*P* = 0.003). Similarly, only 34.5% of patients with DD achieved goal BP at the time of discharge in contrast to 66.7% of patients without DD (*P* = 0.059).

## 4. Discussion

The prevalence of left ventricular hypertrophy (LVH) in our cohort of hospitalized ICH patients was 38%, in keeping with the 40% previously reported in a Japanese cohort [24] and lower than the 80% reported in a Houston cohort [21]. Given the well-described differences in the prevalence of LVH in blacks, non-Hispanic whites, and Hispanic whites, it is possible that this reflects the differing race proportions in each study [25]. It is also possible that advances in cardiac imaging or newer TTE criteria for LVH have reduced false positives.

In our sample, neither the presence of LVH nor the presence of DD on TTE, as a marker for cumulative burden of HTN, was a significant independent predictors of initial ICH volume, IVH on presentation, ICH expansion, or new IVH on follow-up neuroimaging. We did find, however, that the presence of LVH on TTE was associated with increased likelihood that patients would require a minimum of three medications to control their blood pressure at the time of discharge. Neither LVH nor DD was a significant independent predictor of patient outcome in our sample. One possible interpretation of these findings is that neither LVH nor DD is an important prognostic indicator in ICH patients. Given that LVH has been shown to be an independent predictor of vascular events in patients with coronary disease [19], hypertensive patients, and the general population [17, 18], it is unlikely that LVH would not serve as a vascular risk factor in hypertensive ICH patients. But among patients with an outcome related to HTN (ICH), the cumulative burden of HTN may not play a role in neurological consequences, provided HTN is managed per guidelines. It is more likely that our brief cross-sectional study did not allow the time necessary for secondary vascular events that would affect patient outcomes to occur.

The absence of depressed left ventricular systolic function suggests that ICH is not a disease of late HHD, but the significantly higher prevalence of DD compared to LVH has not been previously reported among patients with ICH. Possible explanations include dynamic cardiac changes in the acute setting of ICH and a different pattern of cardiac remodeling in response to sustained HTN in blacks. Repeated TTE assessment would be necessary to determine if the high prevalence of DD seen in patients with ICH is a transient phenomenon or potentially a marker of risk for ICH.

Our study is not without limitations. It is limited by its size and its retrospective nature. Patients with spontaneous supratentorial ICH that present to Tulane University Medical Center may not be representative of all patients with this condition. Despite our efforts to show that there was no difference in patients that received an echocardiogram and patients that did not receive one, the potential for survival bias remains as TTE data was not captured on patients who did not survive the acute period or were not stable enough to undergo a TTE.

In conclusion, we found evidence of either LVH or DD on the majority of our hypertensive ICH patients, with low rates of impaired systolic function. These echocardiographic findings suggest changes in myocardial architecture consistent with the early stages of hypertensive heart disease (HHD), which tend to develop 10 years after first diagnosis of HTN. Detecting HHD in its early stage may make it amenable to intervention that could serve to reduce the risk of subsequent vascular events and improve long-term morbidity and mortality. Further research is needed to investigate the prevalence of LVH in stroke patients and its effect on outcome.

## Figures and Tables

**Table 1:** Comparison of supratentorial deep ICH patients with and without TTE.

	TTE not performed (*n* = 19)	TTE performed (*n* = 47)	*P* value
Age, mean ± SD	61.0 ± 10.4	57.5 ± 13.0	0.067
Gender, % male	52.6%	61.7%	0.497
Race, %			
White	36.8%	21.3%	0.366
Black	63.2%	76.6%	
Asian	0%	2.1%	
NIHSS on admission, median (min–max)	15 (5–40) IQR 9,26	15 (1–40) IQR 6,22	0.291
GCS, median (min–max)	12 (3–15) IQR 6,15	14 (3–15) IQR 10,15	0.191
ICH score, median (min–max)	1 (0–4) IQR 0,2	1 (0–4) IQR 0,2	0.165
Initial volume mL, median (min–max)	10 (1–144) IQR 6,21	12 (0–103) IQR 4,25	0.938
IVH on initial CT, %	63.2%	42.6%	0.129
Length of stay (d), median (min–max)	7 (1–40) IQR 3,18	10 (1–86) IQR 4,18	0.410
mRS on discharge, median (min–max)	4 (2–6) IQR 3,6	4 (1–6) IQR 3,5	0.917
Discharge disposition, %			
Home	15.8%	19.1%	0.559
Inpatient Rehabilitation	42.1%	40.4%	
SNF	5.3%	19.1%	
LTAC	5.3%	6.4%	
Hospice	0%	2.1%	
Death	26.3%	10.6%	
Other	5.3%	2.1%	

NIHSS indicates NIH stroke scale; GCS: Glasgow coma scale; ICH: intracerebral hemorrhage.

**Table 2:** Baseline demographics of supratentorial deep ICH patients with TTE.

	No LVH *n* = 29	LVH *n* =18	No DD *n* = 15	DD *n* = 32
Age, mean ± SD	56.0 ± 13.3	60.1 ± 12.5	54.3 ± 16.6	59.0 ± 58.5
Gender, % male	58.6%	66.7%	66.7%	59.4%
Race, %				
White	24.1%	16.7%	26.7%	18.8%
Black	75.9%	77.8%	73.3%	78.1%
Asian	0%	5.6%	0%	3.1%
NIHSS on admission, median (min–max)	16 (1–40) IQR 8,24	11 (1–24) IQR 4,18	16 (1–31) IQR 5,24	15 (1–40) IQR 6,22
Hypertension (HTN), %	82.8%	88.9%	86.7%	84.4%
Current BP medication use, %	37.9%	61.1%	40.0%	50.0%
Admission SBP†, mean ± SD	182.7 ± 32.3	213.1 ± 38.0	186.3 ± 42.6	198.1 ± 34.7
Admission DBP, mean ± SD	105.8 ± 21.9	117.8 ± 19.5	105.2 ± 20.2	112.9 ± 22.2
Time from LSN to initial CT				
<3 hours	61.5%	58.8%	53.3%	60.0%
3–6 hours	17.9%	0%	20.0%	6.7%
>6 hours	25.0%	41.2%	26.7%	33.3%
Initial volume mL, median (min–max)	14 (0–103) IQR 4,39	10 (1–56) IQR 3,22	12 (1–67) IQR 4,47	12 (0–103) IQR 3,22
Supratentorial ICH, %	100%	100%	100%	100%
ICH location, %				
Basal ganglia	79.3%	55.6%	80.0%	65.6%
Thalamus	20.7%	44.4%	20.0%	34.4%
ICH score, median (min–max)	1 (0–4) IQR 0,2	1 (0–3) IQR 0,1	1 (0–3) IQR 0,2	1 (0–4) IQR 0,2
GCS, median (min–max)	14 (3–15) IQR 10,15	15 (8–15) IQR 12,15	14 (6–15) IQR 10,15	15 (3–15) IQR 10,15
Hydrocephalus, %	41.4%	27.8%	33.3%	37.5%
IVH on initial CT, %	44.8%	38.9%	40.0%	43.8%
CTA performed†, %	69.0%	38.9%	46.7%	62.5%
Spot sign present on CTA, %	10.0% (2/20)	14.3% (1/7)	14.3% (1/7)	10.0%
Dot sign present on CTA, %	15.8% (3/19)	16.7% (1/6)	14.3% (1/7)	16.7% (3/18)
TTE performed, %	100%	100%	100%	100%
LVH, %	0%	100%	40.0%	37.5%
LV size†, mean ± SD (g/m^2^)	90.4 ± 22.3	148.0 ± 41.4	90.5 ± 7.8	112.5 ± 42.3
LVH, %				
Mild	n/a	44.4%	66.7% (4/6)	33.3% (4/12)
Moderate		44.4%	33.3% (2/6)	50.0% (6/12)
Severe		11.1%	0% (0/6)	16.7% (2/12)
Concentric LVH, %	n/a	100%	100% (6/6)	100% (12/12)
Diastolic dysfunction (DD), %	69.0%	66.7%	0%	6.5%
EF < 50%, %	3.7% (1/27)	5.6%	0%	6.5%
EF < 30%, %	0% (0/27)	0%	0%	0%
LV dilated on TTE, %	0% (0/15)	6.3%	0%	6.5%

BP indicates blood pressure; DBP: diastolic blood pressure; SBP: systolic blood pressure; LSN: last seen normal; CTA: computed tomography angiogram; CTA dot and spot signs are foci of high density contrast within the ICH following venous contrast injection (dot < spot) often interpreted as sites of active bleeding; GCS: Glasgow coma scale; IVH: intraventricular hemorrhage; EF: ejection fraction; TTE: transthoracic echocardiogram; LVH: left ventricular hypertrophy; LV: left ventricle.

† *P* < 0.05 for LVH comparisons;

‡ *P* < 0.05 for DD comparisons.

**Table 3:** Treatment and outcome for supratentorial deep ICH patients with TTE.

	No LVH *n* = 29	LVH *n* = 18	No DD *n* = 15	DD *n* = 32
Clot evacuation, %	0%	0%	0%	0%
EVD, %	24.1%	16.7%	13.3%	25.0%
Mannitol, %	20.7%	11.1%	26.7%	12.5%
Received hemostatic agent, %	20.7%	11.1%	20.0%	15.6%
Received activated factor VII, %	3.4%	0%	6.7%	0%
Received vitamin K, %	3.4%	0%	0%	3.1%
Received FFP, %	6.9%	0%	6.7%	3.1%
Received platelets, %	10.3%	11.1%	6.7%	12.5%
Any infectious complication, %	24.1%	27.8%	20.0%	28.1%
Urinary tract infection, %	10.3%	27.8%	13.3%	18.8%
Pneumonia, %	20.7%	11.1%	6.7%	21.9%
Bacteremia, %	6.9%	5.6%	0%	9.4%
DVT, %	0%	0%	0%	0%
NIHSS at 24 hrs, median (range)	19 (1–40) IQR 1,29 *n* = 9	6 (1–27) IQR 1,22 *n* = 5	10 (1–32) IQR 2,28 *n* = 4	16 (1–40) IQR 6,26 *n* = 10
ICH volume at 24 hrs mL, median (min–max)	15 (1–79) IQR 8,52 *n* = 23	10 (1–402) IQR 4,26 *n* = 17	20 (1–402) IQR 9,64 *n* = 11	10 (1–79) IQR 5,26 *n* = 29
New IVH on 24 h CT†, %	0%	16.7%	13.3%	3.1%
ICH volume growth in 24 hrs mL, median (min–max)	0 (−36−23) IQR −1,7 *n* = 23	0 (−4−346) IQR 0,2 *n* = 17	1 (−4−346) IQR −3,7 *n* = 11	0 (−36−23) IQR 0,3 *n* = 29
ICH expansion, %	34.5%	38.9%	40.0%	34.4%
NIHSS at discharge, median (range)	18 (1–42) IQR 4,42 *n* = 10	15 (1–42) IQR 3,42 *n* = 7	29 (1–42) IQR 3,42 *n* = 6	17 (1–42) IQR 4,25 *n* = 11
Length of stay (d), median (min–max)	11 (2–32) IQR 4,19	9 (1–86) IQR 4,18	10 (1–30) IQR 3,14	10 (2–86) IQR 4,19
mRS on discharge, median (min–max)	4 (1–6) IQR 2,5	4 (2–6) IQR 2,5	4 (1–6) IQR 3,5	4 (2–6) IQR 3,5
Discharge disposition, %				
Home	17.2%	22.2%	20.0%	18.8%
Inpatient rehabilitation	41.4%	38.9%	46.7%	37.5%
SNF	24.1%	11.1%	13.3%	21.9%
LTAC	6.9%	5.6%	0%	9.4%
Hospice	0%	5.6%	0%	3.1%
Death	10.3%	11.1%	20.0%	6.3%
Other	0%	5.6%	0%	3.1%

EVD indicates external ventricular drain; DVT: deep venous thrombosis; LTAC: long-term acute care facility; SNF: subacute nursing facility.

† *P* < 0.05 for LVH comparisons;

‡ *P* < 0.05 for DD comparisons.

## References

[1] Caplan LR (1992). Intracerebral haemorrhage. The Lancet.

[2] Broderick JP, Brott T, Tomsick T, Miller R, Huster G (1993). Intracerebral hemorrhage more than twice as common as subarachnoid hemorrhage. Journal of Neurosurgery.

[3] Broderick J, Brott T, Tomsick T (1994). Management of intracerebral hemorrhage in a large metropolitan population. Neurosurgery.

[4] Dennis MS (2003). Outcome after brain haemorrhage. Cerebrovascular Diseases.

[5] Flaherty ML, Haverbusch M, Sekar P (2006). Long-term mortality after intracerebral hemorrhage. Neurology.

[6] Fogelholm R, Murros K, Rissanen A, Avikainen S (2005). Long term survival after primary intracerebral haemorrhage: a retrospective population based study. Journal of Neurology, Neurosurgery and Psychiatry.

[7] Woo D, Sauerbeck LR, Kissela BM (2002). Genetic and environmental risk factors for intracerebral hemorrhage: preliminary results of a population-based study. Stroke.

[8] Flaherty ML, Woo D, Haverbusch M (2005). Racial variations in location and risk of intracerebral hemorrhage. Stroke.

[9] Nilsson OG, Lindgren A, Ståhl N, Brandt L, Säveland H (2000). Incidence of intracerebral and subarachnoid haemorrhage in southern Sweden. Journal of Neurology Neurosurgery and Psychiatry.

[10] Inagawa T, Ohbayashi N, Takechi A (2003). Primary intracerebral hemorrhage in Izumo City, Japan: incidence rates and outcome in relation to the site of hemorrhage. Neurosurgery.

[11] Anderson CS, Chakera TMH, Stewart-Wynne EG, Jamrozik KD (1994). Spectrum of primary intracerebral haemorrhage in Perth, Western Australia, 1989-90: incidence and outcome. Journal of Neurology Neurosurgery and Psychiatry.

[12] Fogelholm R, Nuutila M, Vuorela AL (1992). Primary intracerebral haemorrhage in the Jyvaskyla region, Central Finland, 1985-89: incidence, case fatality rate, and functional outcome. Journal of Neurology Neurosurgery and Psychiatry.

[13] Giroud M, Milan C, Beuriat P (1991). Incidence and survival rates during a two-year period of intracerebral and subarachnoid haemorrhages, cortical infarcts, lacunes and transient ischaemic attacks. The stroke registry of Dijon: 1985–1989. International Journal of Epidemiology.

[14] Kannel WB, Dawber TR, McNamara PM (1965). Vascular disease of the brain—epidemiologic aspects: The Framingham Study. American Journal of Public Health and the Nation's Health.

[15] Cuspidi C, Sala C, Negri F, Mancia G, Morganti A (2012). Prevalence of left-ventricular hypertrophy in hypertension: an updated review of echocardiographic studies. Journal of Human Hypertension.

[16] Cuspidi C, Vaccarella A, Negri F, Sala C (2010). Resistant hypertension and left ventricular hypertrophy: an overview. Journal of the American Society of Hypertension.

[17] Koren MJ, Devereux RB, Casale PN, Savage DD, Laragh JH (1991). Relation of left ventricular mass and geometry to morbidity and mortality in uncomplicated essential hypertension. Annals of Internal Medicine.

[18] Mensah GA, Pappas TW, Koren MJ, Ulin RJ, Laragh JH, Devereux RB (1993). Comparison of classification of the severity of hypertension by blood pressure level and by World Health Organization criteria in the prediction of concurrent cardiac abnormalities and subsequent complications in essential hypertension. Journal of Hypertension.

[19] Levy D, Garrison RJ, Savage DD, Kannel WB, Castelli WP (1990). Prognostic implications of echocardiographically determined left ventricular mass in the Framingham Heart Study. The New England Journal of Medicine.

[20] Albright K, Aysenne A, Chang T (2010). The role of echocar-diography in intracerebral hemorrhage.

[21] Martin-Schild S, Albright KC, Hallevi H (2010). Intracerebral hemorrhage in cocaine users. Stroke.

[22] Dickinson CJ (2001). Why are strokes related to hypertension? Classic studies and hypotheses revisited. Journal of Hypertension.

[23] Morgenstern LB, Hemphill JC, Anderson C (2010). Guidelines for the management of spontaneous intracerebral hemorrhage: a guideline for healthcare professionals from the American Heart Association/American Stroke Association. Stroke.

[24] Yamazaki' T, Yanaka K, Aoki T (2000). Cardiac function estimated by doppler echocardiography in patients with hypertensive intracerebral hemorrhage. Brain and Nerve.

[25] Lee DK, Marantz PR, Devereux RB, Kligfield P, Alderman MH (1992). Left ventricular hypertrophy in black and white hypertensives: standard electrocardiographic criteria overestimate racial differences in prevalence. Journal of the American Medical Association.

